# Influence of Microbiome and Antibiotics on the Efficacy of Immune Checkpoint Inhibitors

**DOI:** 10.7759/cureus.16829

**Published:** 2021-08-02

**Authors:** Priyanka Patel, Arisa Poudel, Sunam Kafle, Manusha Thapa Magar, Ivan Cancarevic

**Affiliations:** 1 Internal Medicine, California Institute of Behavioral Neurosciences & Psychology (CIBNP), Fairfield, USA; 2 Internal medicine, Neurology, California Institute of Behavioral Neurosciences & Psychology (CIBNP), Fairfield, USA

**Keywords:** microbiome, antibiotics, immune checkpoint inhibitors, immunotherapy, anti-pd-1, anti-pd-l1, anti-ctla-4

## Abstract

The human microbiome mainly consists of bacteria and interacts closely with the immune system. Immune checkpoint inhibitors (ICI) are used to treat several types of cancers. Recently, it has been identified that the gut microbiome plays a role in the effectiveness of immunotherapy. This study aims to analyze the effect of microbiome and antibiotics on the effectiveness of ICI in cancer patients and the measures to improve efficacy based on that. A detailed review was conducted on articles published in PubMed and Science Direct in the last five years i.e., 2016 to 2021. A total of 16 articles involving 1293 patients with cancer who were receiving immunotherapy, were deemed eligible to be included in the final review. Data were extracted from the eligible articles and were checked for quality appraisal. All 16 articles revealed the effect of either gut microbiome or antibiotics or both on ICI. Based on our findings, we found that the microbiome enriched in different microorganisms responded differently to the ICI and that antibiotics negatively impacted the effectiveness of ICI. The time at which patients receiving ICI were prescribed antibiotics influenced the effect of ICI. Antibiotics and different microbiome also affected progression-free survival (PFS) and overall survival (OS).

## Introduction and background

The term "human microbiome" was coined by Joshua Lederberg in 2001. The human body consists of 10 to 100 trillion microbial cells that are mainly bacteria, in the gut [1]. A microbiome is a group of microorganisms that interact with each other in a contiguous environment [2]. The microbiome consists of bacteria, archaea, fungi, algae, and small protists [2]. A majority of diverse microbial species are found in the distal gastrointestinal tract [3]. Therefore, the intestinal microbiota is essential in the maturation of host immune responses, providing protection against overgrowth of organisms, providing energy requirements, influencing host-cell proliferation and vascularization, regulating the endocrine function of intestines, neurologic signaling, biosynthesis of vitamins, modifying specific drugs, and removing certain toxins [3].

The purpose of microbiota in the human body is classified into three types: (1) Intestinal dominant bacteria mainly composed of lactic acid bacteria, bacteroids, nitrobacteria, and eubacteria that play a vital role in immunity, nourishment, digestion, and absorption; (2) Commensal bacteria such as *Escherichia coli* (*E. coli*), lactobacilli, and enterococci that have physiologic functions and are pathogenic as well in specific quantities; (3) Passenger bacteria such as Proteobacteria and* Pseudomonas aeruginosa*, which are primarily pathogenic when the intestinal flora is imbalanced but are not pathogenic in smaller quantities [4]. Due to the gut microbiome's close interaction with the immune system, it has recently gained increased attention for its potential role in immunotherapy [5].

Cancer immunotherapy manipulates the host immune system and utilizes the body's immune system mechanisms for cancer treatment [6]. Some of the immune system components used in the development of immunotherapy are cytokines, immune cells, and monoclonal antibodies [6].

The evolution of immune checkpoint inhibitors (ICI) has been a great success in cancer treatment [7]. Immune checkpoint molecules control the host immune system by transmitting immunosuppressive signals to immunocompetent cells [8]. Some common immune checkpoint molecules are cytotoxic T-lymphocyte-associated antigen 4 (CTLA-4), programmed cell death protein/programmed death-ligand 1 (PD-1/PD-L1), and programmed cell death 1 ligand 2 (PD-L2) [7]. They play a role in the suppression of anti-tumor immunity [9]. The main function of ICI is to lessen the immunosuppressive effect in a tumor by targeting immune checkpoint molecules [8]. Antibodies against the immune checkpoint molecules approved for cancer treatment to date are anti-PD-1 antibodies (nivolumab and pembrolizumab), anti-PD-L1 (atezolizumab, avelumab, and durvalumab), and anti-CTLA-4 antibodies (ipilimumab) [8, 9]. The first approved ICI was ipilimumab for patients with advanced melanoma [7].

The response rate of a patient to immunotherapy depends on immune competency, diversity, antigen specificity variation, antigen expression, and the recent identification of the role of gut microbiota [10]. The microbiome plays a role in developing inflammation, the integrity of mucosal immunity, and protecting against pathogens [11]. Side effects of ICI include severe diarrhea and colitis, which suggests the role of the gut microbiome and its influence on therapeutic purpose and toxicity [11].

This systematic review aims to learn about how the gut microbiome influences the effectiveness of ICI and identify strategies to improve the efficacy of immunotherapy.

## Review

Method

Two independent reviewers carried out a rigorous literature review using electronic databases. The Preferred Reporting Items for Systematic Reviews and Meta-Analyses (PRISMA 2020) guidelines were followed for carrying out this systematic review. PubMed and Science Direct were screened for peer-reviewed articles published in the last five years i.e., 2016 to 2021. A search was performed using medical subject headings (MeSH) and keywords combined with Boolean connectors from April 2021 to May 2021.

The search strategy for PubMed was as follows: ("Microbiota/drug effects"[Majr] OR "Microbiota/immunology"[Majr] OR "Microbiota/physiology"[Majr]) OR Microbiome OR “Gastrointestinal microbiome” OR “intestinal flora” OR “bacterial flora” AND ("Immunotherapy/drug effects"[Majr] OR "Immunotherapy/immunology"[Majr] OR "Immunotherapy/pharmacology"[Majr] OR "Immunotherapy/physiology"[Majr] OR "Immunotherapy/therapeutic use"[Majr]) OR Immunotherapy OR “molecular targeted therapy” OR “immunological therapy” AND ("Neoplasms/drug effects"[Majr] OR "Neoplasms/immunology"[Majr] OR "Neoplasms/microbiology"[Majr] OR "Neoplasms/pathogenicity"[Majr] OR "Neoplasms/pathology"[Majr] OR "Neoplasms/pharmacology"[Majr] OR "Neoplasms/physiopathology"[Majr]) OR Cancer OR Neoplasm OR malignancy AND Influence OR effect OR impact

**Figure 1:**
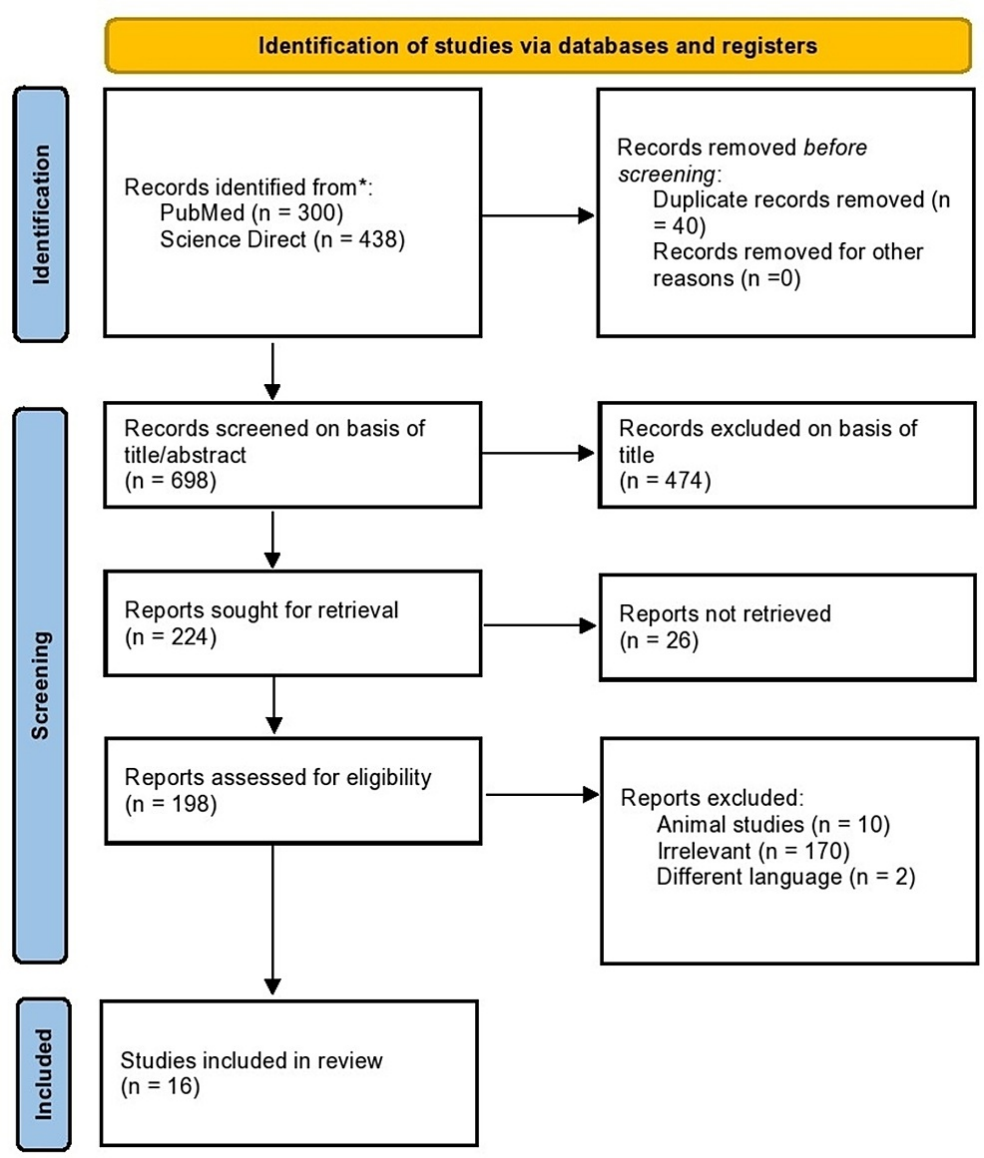
Flowchart of literature review search as per Preferred Reporting Items for Systematic Reviews and Meta-Analyses (PRISMA 2020) guidelines

For Science Direct, the keywords 'immunotherapy' and 'microbiome' were used. Both authors screened each article by title initially. The articles selected based on their title were further evaluated and were included or excluded based on abstract review. A complete article review was done following the abstract review to exclude irrelevant articles. Any duplicate articles that coincided with multiple keywords or MeSH terms were also excluded. Figure 1 shows how the search was conducted as per PRISMA 2020 guidelines [12].

Inclusion Criteria

The search was limited to full-text publications between the years 2016 and 2021, articles published in English, and studies recruiting the adult population (aged more than or equal to 18). We ensured that the studies chosen featured patients who had received at least one ICI (anti-PD-1, anti-PD-L1, or anti-CTLA-4). And our study in turn looked at the effect of either or both microbiome and antibiotics on ICI.

Exclusion Criteria

All studies that were conducted on the children population and animals, case reports and case series, and inaccessible full-text articles were excluded. 

Data Extraction and Study Selection

Two researchers carried out an individual review of the literature. After compiling the results, both researchers compared the data and resolved any conflicts through mutual understanding. 

Results

Initial screening of the electronic database PubMed yielded 300 records, and Science Direct yielded 438 records. After 40 duplicates were removed, the remaining 698 articles were screened by title for relevance. Following that 474 non-relevant articles were excluded. From the remaining 224 articles, 26 articles couldn't be retrieved. Abstracts and full-text of the remaining 198 articles were thoroughly reviewed, and 170 were excluded due to irrelevance, two articles were not in the English language, and 10 were animal studies. Finally, 16 articles were included in the review. Out of the 16, seven were retrospective cohort studies, five were prospective cohort studies, and four were review articles.

Quality Assessment

We carried out a thorough quality assessment for 12 cohort studies using the Newcastle-Ottawa scale and four review articles using a quality appraisal checklist for qualitative studies. Table 1 shows the quality appraisal for cohort studies done by the Newcastle-Ottawa scale.

**Table 1:** Quality appraisal table (Newcastle-Ottawa scale for cohort study)

Author	Selection	Comparability and outcome	Total score
Castello et al. [13]	4	4	8
Tinsley et al. [14]	4	3	7
Zheng et al. [15]	4	4	8
Lui et al. [16]	4	4	8
Pinato et al. [17]	4	4	8
Derosa et al. [18]	4	3	7
Ahmed et al. [19]	4	4	8
Gopalakrishnan et al. [20]	4	4	8
Frankel et al. [21]	4	4	8
Kaderbhai et al. [22]	4	4	8
Thompson et al. [23]	4	4	8
Chaput et al. [24]	4	4	8

Articles with a Newcastle-Ottawa scale score more than or equal to 6/8 and articles with a quality appraisal checklist for qualitative studies score more than or equal to 12/14 were considered high-quality articles.

Articles with a Newcastle-Ottawa scale score between 4-6/8 and articles with a quality appraisal checklist for qualitative studies score between 8-12/14 were considered intermediate quality articles.

Articles with a Newcastle-Ottawa scale score between 1-4/8 and articles with a quality appraisal checklist for qualitative studies score between 1-8/14 were considered low-quality articles.

Out of 12 cohort studies, 10 scored 8/8, and two scored 7/8 on the Newcastle-Ottawa scale. Therefore, all 12 cohort articles were high-quality articles. Out of four review articles, two scored 12/14, and the other two scored 11/14 on the quality appraisal checklist for qualitative studies. And so, two review articles were high-quality articles and the remaining two review articles were intermediate quality articles.

Study Characteristics

The finalized articles were all peer-reviewed articles published in the last five years i.e., 2016 to 2021, with full texts freely available. All articles were in English, and articles in other languages were excluded. Only human studies were included. 

Table 2 shows the data extracted from the studies. Out of 12 cohort studies, seven studies looked for the influence of antibiotics and the gut microbiome on ICI [13, 14, 17-19, 22, 23], and five studies checked for the effect of the gut microbiome on ICI [15, 16, 20, 21, 24].

**Table 2:** Data extraction table NSCLC: Non-small cell lung cancer; M: Male; F: Female; ICI: Immune checkpoint inhibitors; PFS: Progression-free survival; OS: Overall survival; ATB: Antibiotics; BCLC: Barcelona clinic liver cancer; RCC: Renal cell carcinoma; HCC: Hepatocellular carcinoma; SCLC: Small cell lung cancer; ATB 30: Antibiotics within 30 days of starting ICI

Sr. no	Author	Year of Pub	Study design	Population	Sample size	Outcome
1.	Castello et al. [13]	2021	Prospective study	Adult	NSCLC patients - 50 (M - 34, F - 16) (ATB - 20, No ATB - 30)	Antibiotic therapy is associated with poor ICI response, PFS, and a higher tumor burden.
2.	Tinsley et al. [14]	2020	Retrospective study	Adult	Advanced cancer patients - 291 (Metastatic melanoma- 179, NSCLC - 64, RCC- 48) (ATB- 92, No ATB - 199)	Antibiotic use is a negative predictor of PFS and OS in patients with advanced cancer on ICI.
3.	Zheng et al. [15]	2019	Retrospective cohort study	Adult	HCC patients with Barcelona clinic liver cancer (BCLC) stage C - 8	Gut microbiome characteristics may be used for early prediction of the six-month outcome of anti-PD-1 therapy in three to six weeks after treatment initiation in HCC patients.
4.	Lui et al. [16]	2019	Retrospective cohort study	Adult	Advanced lung cancer patients 26 (M - 18, F - 8) (NSCLC - 22, SCLC - 4) (ATB 26)	Intestinal microbiota might be a potential biomarker to help predict diarrhea in patients treated with anti-PD-1 antibodies.
5.	Pinato et al. [17]	2019	Prospective study	Adult	Cancer patients - 196 (M - 137, F - 59) (NSCLC - 119, melanoma - 38, other tumor type - 39) (ATB - 196)	Antibiotic treatment before ICI therapy is associated with worse treatment response and OS.
6.	Derosa et al. [18]	2018	Retrospective cohort study	Adult	RCC patients - 121 (M - 80, F - 41) (ATB 30 - 16, No ATB - 105) (NSCLC patients - 239) (M - 118, F - 121) (ATB 30 - 48, No ATB - 191)	Antibiotics were associated with decreased benefits from ICI in RCC and NSCLC.
7.	Ahmed et al. [19]	2018	Retrospective cohort study	Adult	Advanced cancer patients - 60 (M - 35, F - 25) (ATB - 17, No ATB - 43)	The use of broad-spectrum antibiotics while receiving ICI treatment is associated with a poor response rate and shorter PFS.
8.	Gopalakrishnan et al. [20]	2018	Prospective study	Adult	Metastatic melanoma patients - 89	Patients with favorable gut microbiome have a better response to anti-PD-1 immunotherapy due to increased antigen presentation and improved effector T cell function.
9.	Frankel et al. [21]	2017	Prospective study	Adult	Melanoma patients - 39	Commensal flora could be different in responders depending on ICI.
10.	Kaderbhai et al. [22]	2017	Retrospective study	Adult	NSCLC patients - 74 (M - 60, F - 14) (ATB - 15, No ATB - 59)	No effect of antibiotics on the efficacy of nivolumab
11.	Thompson et al. [23]	2017	Retrospective study	Adult	Metastatic NSCLC patients - 74 (ATB - 18, No ATB - 56)	Antibiotic use within six weeks of starting ICI leads to inferior PFS and OS, which suggests a link between antibiotic altering gut microbiome and efficacy of ICI.
12.	Chaput et al. [24]	2017	Prospective study	Adult	Metastatic melanoma patients - 26	Gut microbiota rich in *Faecalibacterium* and other Firmicutes has a better clinical response to ipilimumab and more frequent episodes of ipilimumab-induced colitis.

A total of 1293 patients were included in this study with different types of malignancy at different stages requiring immunotherapy. Of the 1293 patients included in the 16 selected publications, 448 patients received antibiotics, and the remaining 845 didn't receive antibiotics. Majority of the patients had lung cancer (n= 646), melanoma (n= 371) and RCC (n= 169). All the patients at least received an anti-PD-1 or anti-PD-L1 or anti-CTLA-4 treatment.

Overall, the chosen studies showed a negative impact of antibiotics on the response of ICI. Studies have also demonstrated the effect of the gut microbiome on the efficacy of ICI. A retrospective study performed by Kaderbhai et al. didn't show any impact of antibiotics on the effectiveness of nivolumab [22]. Another prospective study conducted by Chaput et al. demonstrated a better clinical response to ICI if the gut microbiome is enriched in *Faecalibacterium* and other Firmicutes [24].

Discussion

Effect of Antibiotics on the Microbiome

Antibiotics have helped in the morbidity and mortality of various fatal infections, but they have a speedy and long-lasting effect on gut microbiota composition [13, 25]. Antibiotics alter the gut microbiome by causing loss of diversity, metabolic capacity changes, loss of vital taxa, and decreasing colonization resistance against invasive pathogens [25]. These changes are named gut dysbiosis [25]. Gut dysbiosis occurs within days of exposure, and it may take >6 weeks to recover after broad-spectrum antibiotic treatment [14]. A decrease in gut microbiota was noted lasting four years after the seven-day treatment of *Helicobacter pylori* with clarithromycin, metronidazole, and omeprazole [14]. Antibiotics also impair the response of cytotoxic T-cells against cancer [17]. Due to the gut dysbiosis caused by antibiotics, their use negatively influences immune response against cancer cells [13].

Antibiotics help in curing mild and severe infections but at the same time cause dysfunction in the gut microbiome. It has a chronic harmful effect on the microbiome by decreasing its diversity. Gut microbiome dysfunction, in turn, causes a damaging effect on the immune response.

Effect of the Microbiome on ICI Efficacy

Baseline gut microbiota is an important marker for the response of treatment and anti-CTLA-4 treatment-induced colitis, according to a study by Chaput et al. [24]. Their study showed gut microbiome rich in the Bacteroides genus had an inadequate response to anti-CTLA-4 treatment and remained free of colitis [24]. Patients who had a gut microbiome rich in Firmicutes had longer progression-free survival (PFS) and overall survival (OS) and had increased occurrence of anti-CTLA-4 induced colitis [24]. This evidence is also supported by a study conducted by Gopalakrishnan et al., which showed patients having enhanced anti-tumor immune response if they have a favorable gut microbiome consisting of high diversity and enriched in Ruminococcaceae/*Faecalibacterium* [20]. This enhanced response is due to increased antigen presentation and enhanced effector T cell function [20]. Patients with an unfavorable gut microbiome i.e. a microbiome with less diversity and a high abundance of Bacteroidales, have decreased anti-tumor immune response [20]. This reduced response is due to weak antigen presentation and inadequate intratumoral lymphoid and myeloid infiltration [20]. A metagenomic shotgun sequencing and metabolomic profiling performed by Frankel et al. showed that different ICI responders have different microbiomes [21]. This study found that patients responding to a combination of ipilimumab plus nivolumab had a gut microbiome enriched in *Faecalibacterium prausnitzii,* *Bacteroides thetaiotamicron*, and *Holdemania filiformis* [21]. Pembrolizumab responders had gut microbiome enriched in *Dorea formicigenerans* [21]. ICI responders for all types of treatment were found to have microbiome enriched in *Bacteroides caccae* [21].

Patients who respond to anti-PD-1 immunotherapy have increased taxonomic diversity and enriched genes than the non-responders of anti-PD-1 immunotherapy [15]. Before immunotherapy treatment, both the responders and non-responders have an abundance of Bacteroidetes, followed by Firmicutes and Proteobacteria similar to a healthy adult [15]. After the treatment initiation, responders have stable microbial composition, while non-responders have increased Proteobacteria [15]. This suggests that gut microbial diversity is associated with drug efficacy and disease prognosis [15]. It was also found that oral administration of *Bifidobacterium* can improve the efficacy of anti-PD-L1 immunotherapy [15].

It is very common for patients receiving immunotherapy to have diarrhea as an immune-related side effect [16]. Intestinal microbiota plays a vital role in immune-related diarrhea in patients receiving immunotherapy [16].

In summary, the intestinal microbiome plays a vital role in response to ICI. And ICI responders have microbiome enriched in different microbes compared to non-responders. Microbiome enriched in Firmicutes, Ruminococcaceae/*Faecalibacterium*, *Holdemania filiformis*, *Dorea formicogenerans*,* Bacteroides thetaiotamicron*, and *Bacteroides caccae* has a beneficial effect on the efficacy of ICI due to increased antigen expression. An enriched microbiome led to longer PFS and OS, but at the same time, it increased ICI-related colitis. Microbiome rich in Bacteroides genus has an inappropriate response to ICI due to decreased antigen expression and has a lesser occurrence of ICI-related colitis. Non-responders of ICI have increased Proteobacteria compared to stable microbial composition in responders.

Effect of Antibiotics on ICI Efficacy

Antibiotics modify the gut microbiome, and clinical studies have emphasized the negative effect of antibiotics in patients treated with ICI [13]. Antibiotics are associated with decreased PFS in patients on ICI [13]. A study conducted by Tinsley et al. showed a decrease in PFS and OS in patients on ICI receiving antibiotics [14]. Multiple and lengthy antibiotic use results in inadequate clinical outcomes [14]. Antibiotic therapy 30 days before initiation of ICI has shown poor response rate and survival in cancer patients on ICI [17]. However, concurrent use of antibiotics and ICI was not associated with poor response or decreased OS [17]. A study conducted by Derosa et al. showed there was an absence of immune-related colitis when the fecal sample was enriched in Bacteroidetes and depleted in Firmicutes [18]. This study also showed increased progressive disease and poor PFS and OS in patients receiving ICI who were treated with antibiotics [18]. The use of narrow-spectrum antibiotics did not affect the response rate, while broad-spectrum antibiotics decreased the response rate [19]. It was also found that antibiotics received two weeks before and after starting ICI had extended time to respond, lower response rate and PFS than patients who didn't receive antibiotics during that period [19]. Thompson et al. conducted a study that showed patients who received antibiotics within six weeks of starting ICI had shorter PFS and OS [23]. Contrary to these findings, a study conducted by Kaderbhai et al. showed antibiotics didn't have any effect on nivolumab in lung cancer patients [22].

Overall, antibiotics have a damaging effect on the response of ICI due to their negative impact on the gut microbiome. The use of several and prolonged broad-spectrum antibiotics leads to an inadequate ICI response. Antibiotic use is also associated with inferior PFS and OS in some cancer patients receiving ICI. The administration of antibiotics during different phases of ICI also plays a role in clinical outcomes in patients receiving ICI.

An intact gut microbiome is required for an adequate immunotherapy effect. Antibiotics should be used very carefully in cancer patients on immunotherapy to prevent drug resistance and inadequate response to ICI. Manipulation of the microbiome should be considered for ICI therapy.

Limitations

Some limitations of the study are that there have not been enough studies evaluating the efficacy of ICI by other products altering the gut microbiome, such as probiotics. We didn't take other factors affecting gut microbiomes like diet, exercise, and age into consideration. The search was limited to articles in the English language, which might have missed some relevant articles. The search was limited to two databases. However, on performing a thorough literature review of these two databases, studies that feature over 1000 patients were included and this was deemed an adequate sample size to derive a conclusion.

## Conclusions

In this systematic review, we evaluated how the microbiome and antibiotics affect the efficacy of immunotherapy. The effectiveness of immunotherapy depends on the type of microbiome present in the gut. Antibiotics were found to influence the gut microbiome negatively, hence decreasing the efficacy of immunotherapy. This proves that a stable microbiome needs to be maintained for increased effectiveness of immunotherapy. It is recommended that antibiotics be used very judiciously in cancer patients on ICI. The use of microbiome sequencing in the future can be a helpful tool in predicting ICI response due to variation in the microbiome in responders and non-responders of ICI.
